# Supplementary figures and images for: The Drosophila FUS ortholog cabeza promotes adult founder myoblast selection by Xrp1-dependent regulation of FGF signaling
Source: PLoS Genet. 2020 Apr 17;16(4):e1008731. doi: 10.1371/journal.pgen.1008731 (PMC7190187; doi:10.1371/journal.pgen.1008731)

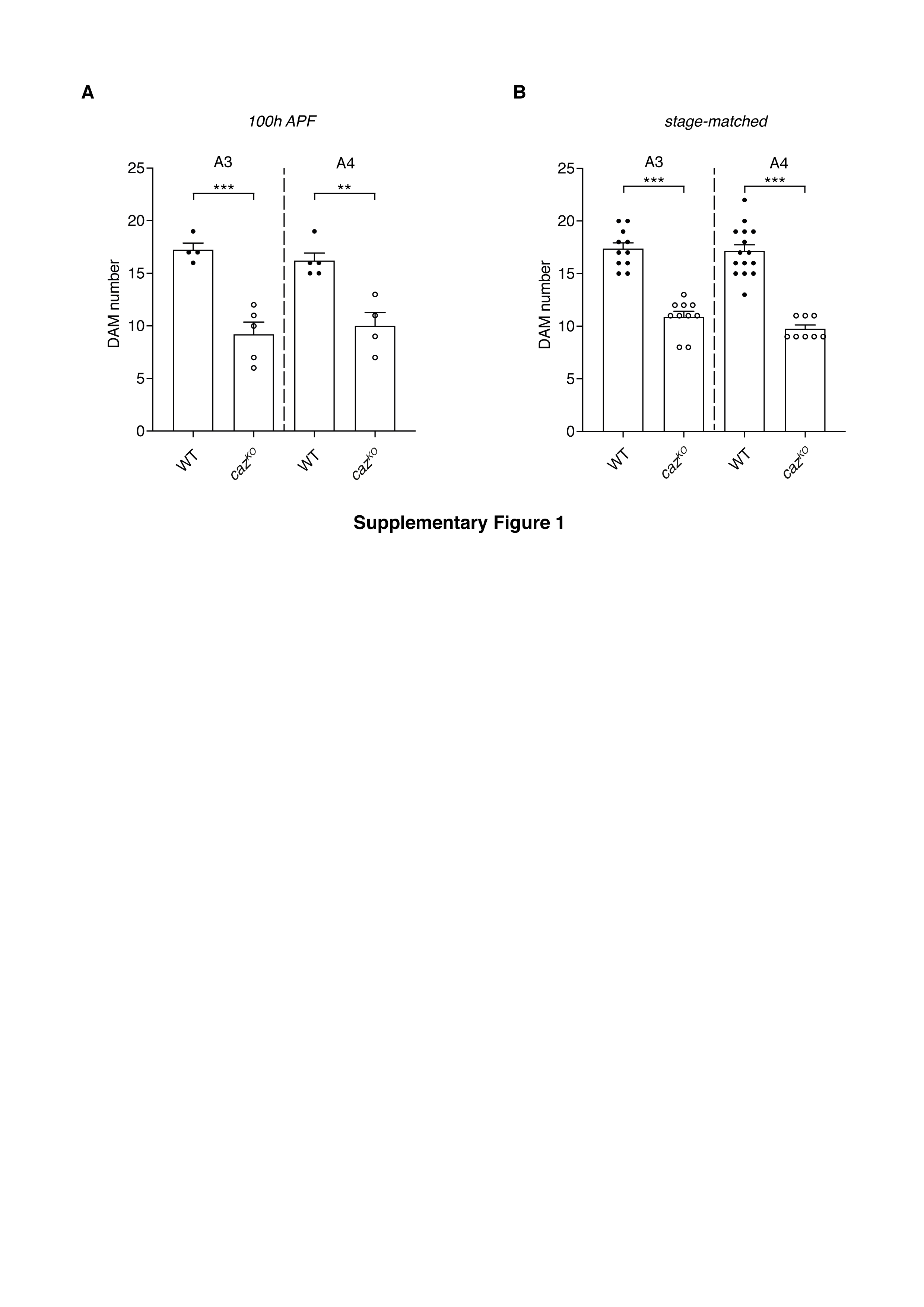

Supplement: S1 Fig — A,B, Quantification of DAM number in segments A3 and A4 of either age-matched (100 h APF, A) or stage-matched (P15: twitching legs, B) cazKO versus WT male pupal filets immunostained for actin. Unpaired t-test (A, B-A3) and t-test with Welch’s correction (B-A4); **p<0.01, ***p<0.001; n(A) = 4 WT versus 5 cazKO (A3), 5 WT versus 4 cazKO (A4); n(B) = 11 WT versus 10 cazKO (A3), 15 WT versus 8 cazKO (A4). Average ± SEM. (TIF) [file pgen.1008731.s001.tif]

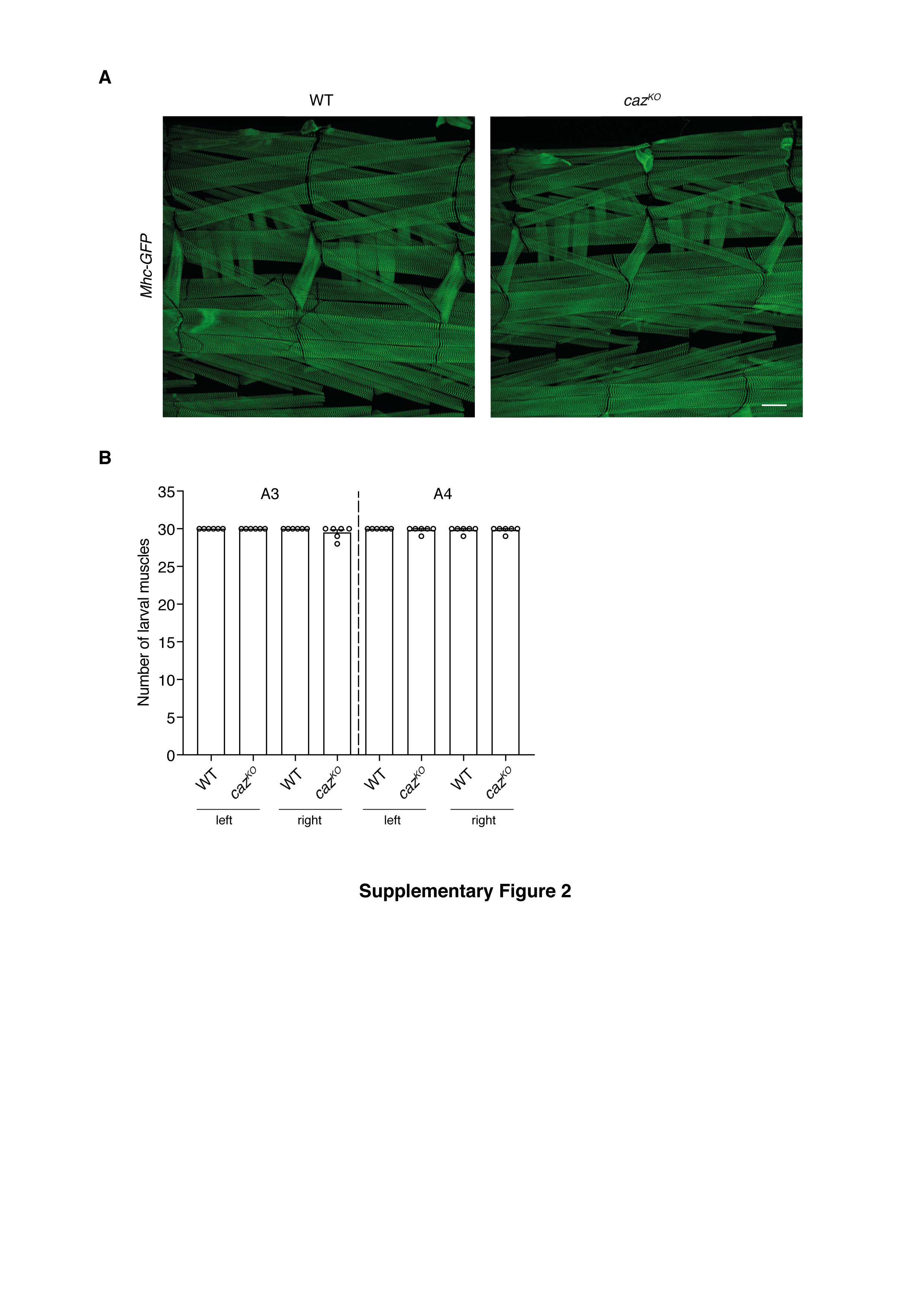

Supplement: S2 Fig — A, A transgenic line in which the Myosin heavy chain gene is GFP-tagged (Mhc-GFP) allowed for visualization of muscles in abdominal segments A3 and A4 of WT (+/Y; Mhc-GFP,his-RFP/+) and cazKO (cazKO/Y; Mhc-GFP,his-RFP/+) third instar larvae. Scale bar: 100μm. B, Quantification of larval muscle number in segments A3 and A4. Mann-Whitney test; n = 6 per genotype. Average ± SEM. (TIF) [file pgen.1008731.s002.tif]

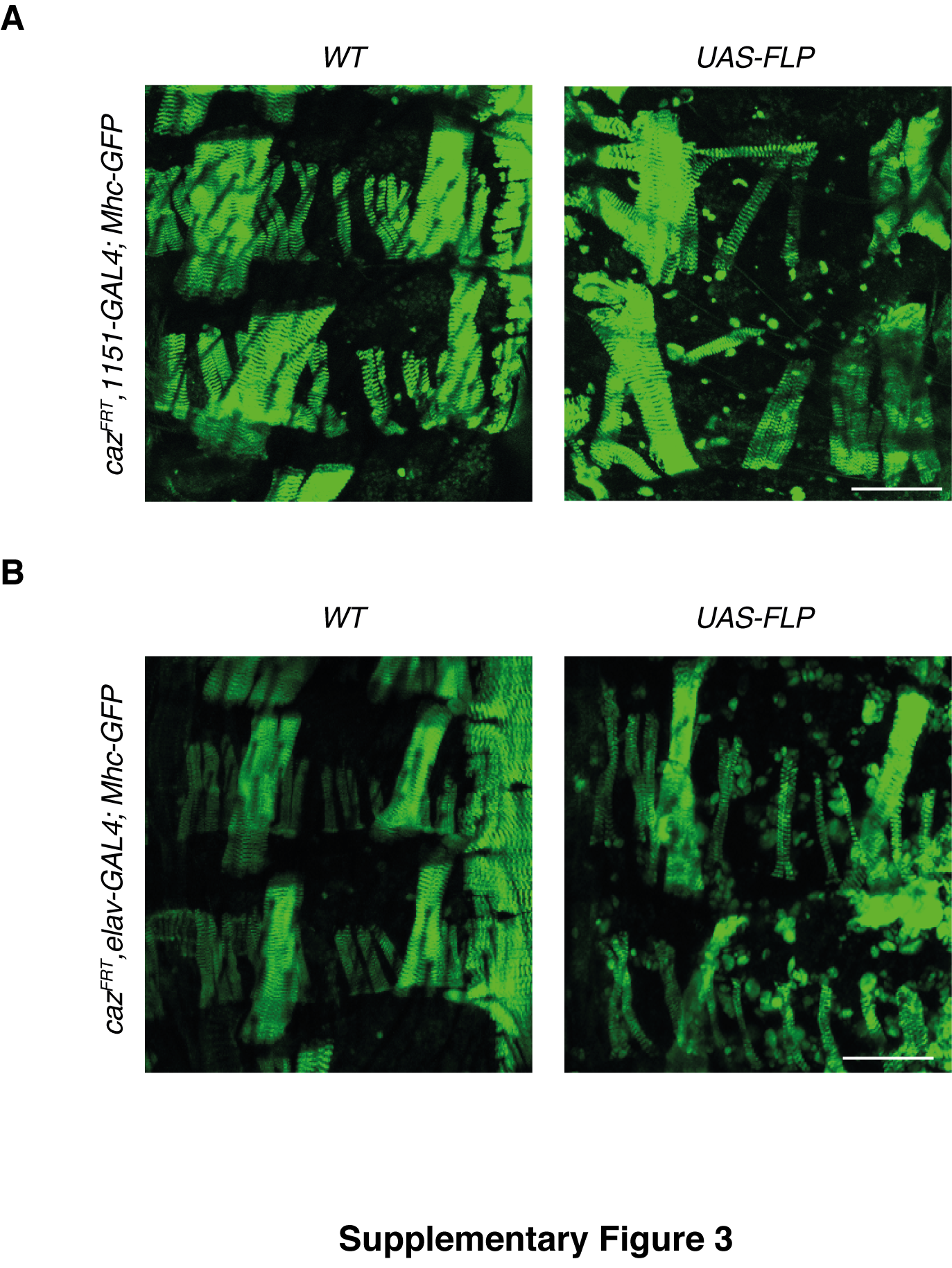

Supplement: S3 Fig — A, Representative images of DAMs in abdominal segments A3 and A4 of 96 h APF pupae in which caz was selectively inactivated in adult myoblasts (cazFRT,1151-GAL4/Y; Mhc-GFP/UAS-FLP, right panel) as compared to the relevant control (cazFRT,1151-GAL4/Y; Mhc-GFP/+, left panel). Scale bar: 100μm. B, Representative images of DAMs in abdominal segments A3 and A4 of 96 h APF pupae in which caz was selectively inactivated in neurons (cazFRT,elav-GAL4/Y; Mhc-GFP/UAS-FLP, right panel) as compared to the relevant control (cazFRT,elav-GAL4/Y; Mhc-GFP/+, left panel). Scale bar: 100μm. (TIF) [file pgen.1008731.s003.tif]

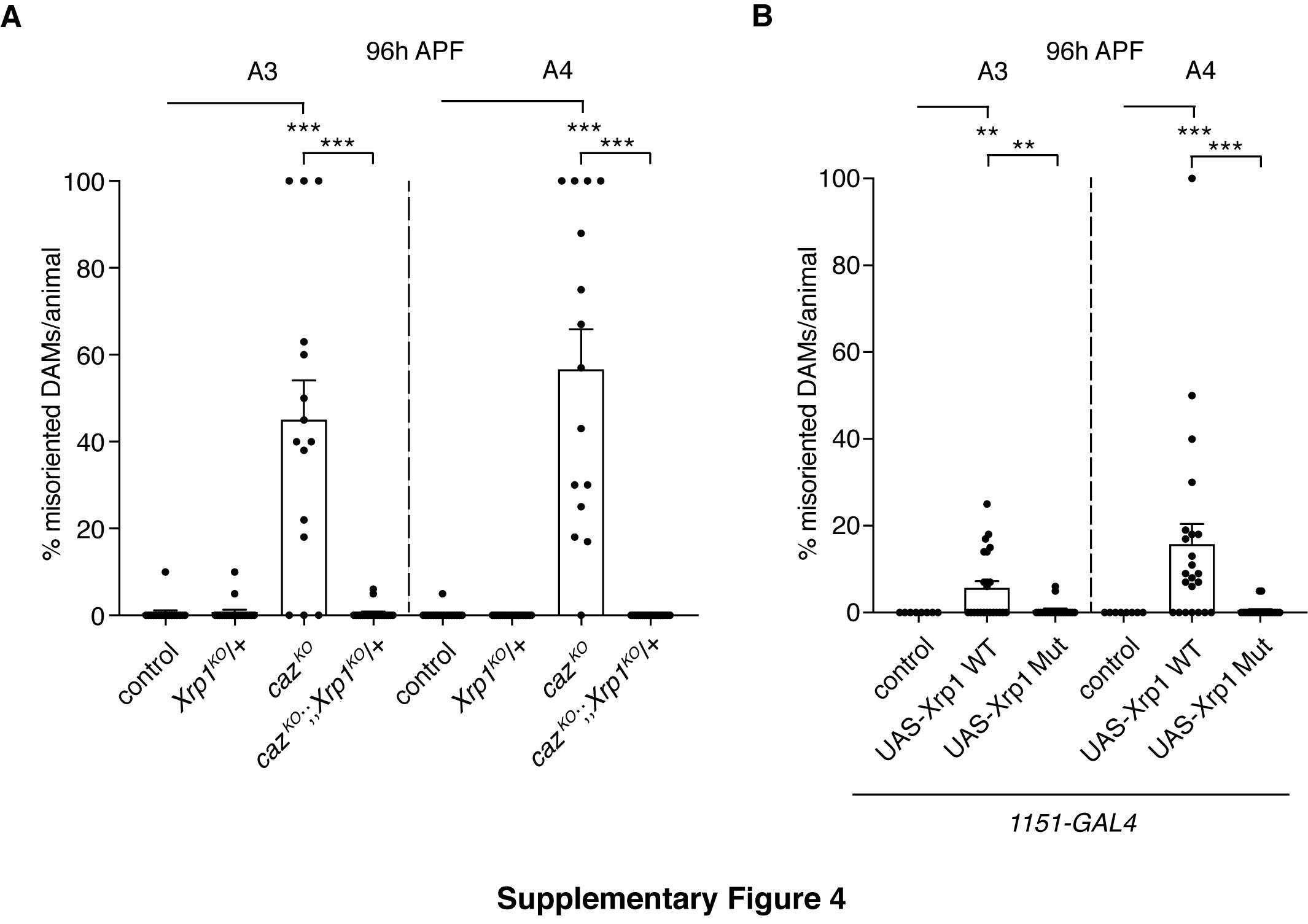

Supplement: S4 Fig — A, Percentage of misoriented DAMs per animal in segments A3 and A4 of 96 h APF cazKO pupae that are heterozygous for Xrp1 (cazKO/Y; Mhc-GFP,his-RFP/+; Xrp1KO/+) as compared to the relevant control genotypes (+/Y; Mhc-GFP,his-RFP/+ // +/Y; Mhc-GFP,his-RFP/+; Xrp1KO/+ // cazKO/Y; Mhc-GFP,his-RFP/+). One sample Wilcoxon signed rank test to compare all genotypes to control, and cazKO to cazKO; Xrp1KO/+; ***p<0.0005; n = 15 per genotype. Average ± SEM. B, Percentage of misoriented DAMs per animal in segments A3 and A4 of 96 h APF male pupae that selectively overexpress Xrp1 in adult myoblasts (1151-GAL4), either as WT protein or with a subtle mutation that disrupts the DNA-binding capacity of the AT-hook motif (Mut), as compared to driver-only control. One sample Wilcoxon signed rank test to compare all genotypes to control and UAS-Xrp1 WT to UAS-Xrp1 Mut; **p<0.01, ***p<0.0001; n = 8 control, 23 UAS-Xrp1 WT, 20 UAS-Xrp1 Mut. Average ± SEM. (TIF) [file pgen.1008731.s004.tif]

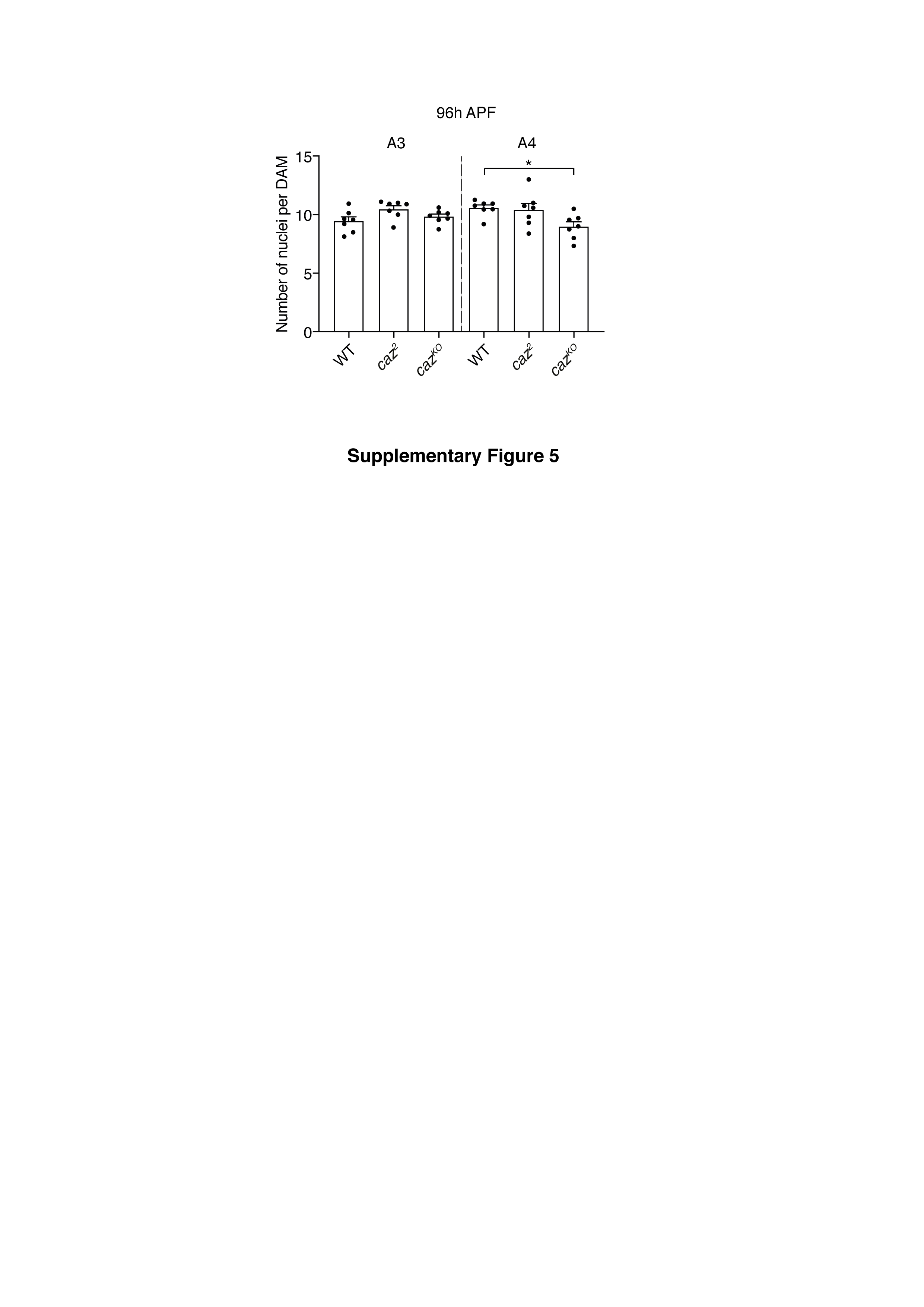

Supplement: S5 Fig — The average number of nuclei per DAM was determined in abdominal segments A3 and A4 of 96 h APF WT, caz2, and cazKO pupae that carried Mhc-GFP and his-RFP transgenes to visualize muscles and nuclei, respectively. Ordinary one-way ANOVA with Dunnett’s post test; *p<0.05; n = 7 per genotype. Average ± SEM. (TIF) [file pgen.1008731.s005.tif]
